# Successful Challenge With Brexpiprazole for Idiopathic Hypersomnia in a Patient With Bipolar Disorder: A Case Report

**DOI:** 10.7759/cureus.53182

**Published:** 2024-01-29

**Authors:** Yuka Shimada, Yasunori Oda, Shintaro Shibata, Yuki Hirose, Tsuyoshi Sasaki

**Affiliations:** 1 Department of Psychiatry, Chiba University Graduate School of Medicine, Chiba, JPN; 2 Department of Child Psychiatry, Chiba University Hospital, Chiba, JPN

**Keywords:** serotonin dopamine activity modulator (sdam), brexpiprazole, methylphenidate (mph), hypersomnolence, narcolepsy, idiopathic hypersomnia (ih)

## Abstract

We describe a 32-year-old Japanese female with hypersomnia and bipolar disorder. She had developed hypersomnia and sleep attacks in her teens. She was misdiagnosed with narcolepsy at a neurology department and then received methylphenidate (MPH) for many years. After giving birth, she developed postpartum depression and suffered from mood swings and irritability. Following 10-year treatment with methylphenidate, she experienced MPH-induced psychosis when she was in a manic state. Her psychosis improved rapidly with the cessation of methylphenidate. Furthermore, brexpiprazole treatment ameliorated her manic symptoms and hypersomnolence. Post-discharge, she was diagnosed with idiopathic hypersomnia based on nocturnal polysomnography and a multiple sleep latency test. This case indicates that brexpiprazole as a serotonin dopamine activity modulator might provide therapeutic effects against not only the patient's manic symptoms but also idiopathic hypersomnia.

## Introduction

Sleep disorder is a major complaint in many psychiatric scenarios. Insomnia is a more common symptom than hypersomnia among individuals with a psychiatric disorder, especially among those with depression. However, estimates of the prevalence of hypersomnia in patients with depression range from 9% to 26% [1]. Idiopathic hypersomnia (IH) is considered a debilitating neurologic sleep disorder and is classified as a central disorder of hypersomnolence, including narcolepsy. Cases of central hypersomnia are thought to lie along a spectrum, and compared to narcolepsy type 1 and type 2, IH has the lowest rate (nearly 82%, 13%, and 5%, respectively) [2]. It has been reported that individuals with IH experience more anxiety and depression compared to those without IH [3]. Patients with IH often describe experiencing autonomic symptoms such as headaches, temperature dysregulation, and fainting more frequently as compared to healthy individuals [4]. Idiopathic hypersomnia can thus negatively affect many aspects of daily life.

The following diagnostic criteria for IH are provided in the International Classification of Sleep Disorders (ICSD) third edition [5]: Overwhelming desire to sleep and falling asleep during the day that continues daily for at least three months; absence of cataplexy; a multiple sleep latency test (MSLT) result with a mean sleep latency ≤8 min or a total sleep time ≥660 min on 24-hour polysomnography (PSG) or wrist actigraphy; ruling out of sleep deprivation; and the symptoms of hypersomnia or MSLT findings not being better explained by other sleep disorders, physical or mental illness, or drug or substance use.

Non-pharmacological treatments for IH such as prolonging sleep times, scheduled naps, and behavioral therapy are not generally effective, but pharmacological therapy can be effective [2]. Regarding pharmacotherapy, the American Academy of Sleep Medicine clinical practice guidelines recommend that clinicians use modafinil for the treatment of IH in adults. Other options for treating IH such as methylphenidate (MPH), clarithromycin, and sodium oxybate have a conditional recommendation [6]. However, the approval of modafinil for IH in Europe was rescinded in 2011 after a re-evaluation found insufficient evidence to support the indication for its use and after reports of rare severe skin reactions [7]. Unfortunately, most individuals with IH still cannot function well in many areas of their lives [4].

Methylphenidate (MPH), which is approved for the treatment of narcolepsy in Japan, acts as a dopamine and catecholamine reuptake inhibitor, resulting in an enhancement of dopaminergic signaling. It is thus likely that MPH may increase the risk of the development of psychosis. There are also several case reports that psychostimulant medications could have antimanic effects. Hegerl et al. proposed the "vigilance regulation model of mania," which contends that psychostimulants could be a preferable treatment option for acute mania in light of their beneficial effects on attention-deficit hyperactivity disorder (ADHD) [8]. Viktorin et al. noted that patients with bipolar disorder receiving MPH monotherapy displayed an increased rate of manic episodes within ~6 months of the monotherapy's introduction, whereas the risk of mania was lower for patients taking mood stabilizers concomitantly. They thus concluded that careful assessment is necessary to rule out bipolar disorder [9]. Taking the above-described findings together, it appears that clinicians probably need to prescribe psychostimulants for patients with bipolar disorder after a thorough consideration of the risk of worsening psychiatric symptoms.

We present the case of our patient with IH and comorbid bipolar disorder who experienced MPH-induced psychosis when she was in a manic state. Her psychotic symptoms rapidly improved with the discontinuation of MPH. In addition, the administration of brexpiprazole (BRX) resolved not only the patient's manic symptoms but also the symptoms of IH.

## Case presentation

The patient was a 32-year-old Japanese female with bipolar disorder who was suffering from hypersomnia. At the age of 18, she began to experience hypersomnia and sleep paralysis. She fell asleep and napped more frequently, and she eventually visited a Department of Neurology. After several examinations (details unknown), she was diagnosed with narcolepsy and received immediate-release MPH 40 mg. She married at the age of 22 and became pregnant at 23. After giving birth, she developed a tendency toward depression and anxiety. Although her attending gynecologist diagnosed postpartum depression, the patient did not return for a follow-up visit. Thereafter, she became aware of mood swings. When she was depressed, she would stay at home and lie in bed all day, but when she became hypomanic, she would cut back on sleep and engage in a variety of tasks. She became euphoric sometimes and then even more overactive. Since that time, the symptoms of bipolar disorder have become more prominent.

At 25 years old, the patient changed her residence and visited a department of psychiatry for hypersomnia, and MPH was prescribed for narcolepsy again. At the age of 27, she was divorced and had started to live on her own. Since her visits to healthcare facilities were irregular and her adherence to the oral MPH fluctuated, her symptoms of hypersomnolence also fluctuated. At the age of 28, she became a welfare recipient. Counseling and support from a community service were started, and during these interventions the staff witnessed the patient fall asleep in front of them on many occasions. Once she fell asleep, she did not wake up easily. In addition, her emotional highs and lows became more pronounced. The community service staff thus decided to visit her more frequently.

In January at the age of 30, the patient was diagnosed with progressive breast cancer (T4b N1 M0 c Stage ⅢB). From that time, she began to suffer mood swings and irritability more frequently. She was referred to our hospital's department of mammary surgery. When she began to receive an explanation about the results of her disease, she suddenly burst into tears, shouted, and irritably paced around the room. Because she became too excited to listen to the explanation, she was referred to our Department of Psychiatry. We diagnosed her with bipolar depression and introduced aripiprazole 3 mg. However, she stopped taking this medication due to akathisia. We thus prescribed valproate sodium 400 mg. She continued to see her original psychiatrist irregularly to obtain and take MPH. In August, after a 5-fluorouracil/epirubicin/cyclophosphamide 4 cycle followed by a weekly paclitaxel 4 cycle, she underwent a left total mastectomy with axillary lymph node dissection. Since then, her breast cancer has not recurred until now.

In February, at the age of 31, the patient gradually experienced mood elevation, grandiose delusions, and flights of ideas. At our medical office, she berated a female psychiatrist because the psychiatrist declined to prescribe benzodiazepine for her. Thereafter, the patient continued to be in a manic state. Furthermore, she became paranoid and considered her caregiver an enemy due to persecutory delusions. In August, she eventually brandished a knife at her caregiver. Police thus brought her to our hospital, and she was involuntarily admitted with the mayor's consent. Her physical examination, blood analysis, and brain MRI findings did not exhibit any significant alterations.

As a result of focused history-taking, we knew that the patient had repeatedly taken overdoses of MPH before going out. Therefore, we supposed that her persecutory delusions were induced by MPH. Considering that MPH may cause psychotic symptoms, we thus discontinued MPH on the day of the patient's admission. We also administered the Japanese version of the Epworth Sleepiness Scale (JESS™) [10], and she scored 21 points. On the second day of her admission, her psychotic symptoms were observed to have rapidly reversed. Although her psychotic symptoms were significantly ameliorated by the discontinuation of the MPH, we speculated that a continuous regimen of psychotropic medication would improve the patient's hypersomnia and manic state. Considering a report that treatment with the dopamine partial agonist aripiprazole ameliorated hypersomnolence in a case of narcolepsy [11], we decided to introduce brexpiprazole (BRX) 1 mg for her instead of aripiprazole, which had induced akathisia. Thereafter, her mood was gradually stabilized and sleep attacks did not occur.

On the first, second, fifth, and twenty-second days after admission, the patient's Young Mania Rating Scale (YMRS) [12] and Positive and Negative Syndrome Scale (PANSS) [13] scores were 50, 20, 18, and 15 points, and 120, 50, 40, and 35 points, respectively. On the twenty-first day, we decided to stop the patient's BRX and follow her closely with valproate sodium 800 mg monotherapy because she had described experiencing a strong thirst while taking BRX. However, she gradually became manic and experienced sleep paralysis and hypersomnolence again on the twenty-fifth day. We thus reintroduced BRX 1mg together with the traditional Japanese 'Kampo' medicine byakkokaninjintou, which is used for unusual thirst. After taking byakkokaninjintou, she no longer complained of thirst. Furthermore, the patient's mood then stabilized and sleep attacks did not occur again.

When the patient was discharged on the thirtieth-day post-admission, her Global Assessment of Functioning (GAF) [14] score improved to 60 points from 20 points at admission. Over eight months have passed since her discharge; after two weeks of psychotropic withdrawal, she underwent a nocturnal PSG examination and completed the MSLT. The PSG showed an apnea-hypopnea index (AHI) of 5.2, the lowest SpO2 at 92%, and an arousal index of 15.8. The MSLT showed a mean sleep latency (MSL) at 7.25 min and no sleep-onset rapid eye movement (REM)-sleep periods (SOREM). An HLA haplotyping test revealed that the patient did not carry HLA DQB1*0602. Based on these results, the patient was diagnosed with IH, not narcolepsy. Her JESS™ score at that time had dropped to 13 points. After discharge from our hospital, she stopped seeing her doctor for a prescription for MPH. While she continues to make outpatient visits to our hospital, she never experienced another sleep attack.

## Discussion

Our patient's abuse of MPH had induced psychosis in her manic state, and her psychotic symptoms improved rapidly after the MPH was discontinued. Treatment with BRX ameliorated not only her manic symptoms but also her hypersomnolence. Since the patient continued to be in a hypomanic state after the discontinuation of MPH, it was appropriate to diagnose her as having IH and comorbid bipolar disorder rather than solely MPH-induced psychosis. To our knowledge, this is the first published report describing the anti-hypnotic and anti-manic effects of BRX on a patient with IH and comorbid bipolar disorder.

There is a large body of literature showing that pharmacotherapy for hypersomnolence is not associated with the risk of psychotic disorders [15]. In addition, anti-manic effects of psychostimulants have been reported in several case reports and case series. From this perspective, Kluge et al. investigated the effects of an initial treatment with MPH on acute mania in a multicenter, randomized, double-blind, placebo-controlled clinical trial [8]. Although they did not observe the effectiveness of MPH for acute mania, their results indicated that MPH was well tolerated and safe. In our patient's case, however, the use of MPH clearly induced psychosis when she was in a manic state. It is thus preferable that a patient's comorbidity with psychotic disorders should be taken into consideration by psychiatrists before they prescribe psychostimulants to patients. It is also apparent that psychiatrists may need to prescribe an antipsychotic or mood stabilizer adjunctively with a psychostimulant when they encounter an IH patient with bipolar disorder or schizophrenia as revealed by the patient's history.

Brexpiprazole is a serotonin-dopamine activity modulator (SDAM) that acts as a partial agonist at dopamine D2/3 and 5-HT1A receptors; it acts as an antagonist at 5-HT2A, 5-HT2C, 5-HT7, and noradrenaline alpha1B/2C receptors [16,17]. In light of its multifunctional profile, BRX was also approved in the USA as an adjunctive treatment with antidepressants for patients with treatment-resistant major depression. Given that aripiprazole has successfully treated narcolepsy or isolated cataplexy [11,18], it is possible that the partial agonistic effects of aripiprazole on dopamine D2 receptors could cause the alleviation of hypersomnolence. BRX is also thought to have a dopamine D2 receptor partial agonistic function, with approximately two-thirds as much signal strength as aripiprazole. BRX has been observed to exert more effects on the serotonin system as compared to aripiprazole. For example, it was proposed that the activation of 5-HT1A and the antagonization of 5-HT2A receptors induce a release of dopamine. The 5-HT7 receptors are suspected to be closely involved in the regulation of sleep and circadian rhythms independent of dopamine [19]. Specifically, 5-HT7R KO mice spent less time in and demonstrated less frequent episodes of REM sleep [20]. In fact, a clinical study revealed that the blockade of 5-HT7 receptors increased healthy volunteers' REM latency and decreased their REM sleep duration [21]. It is thus likely that BRX's partial agonistic effects on dopamine D2 and 5-HT1A receptors and antagonistic effects on 5-HT2A and 5-HT7 receptors may contribute to the amelioration of hypersomnolence.

Regarding treatments for patients with hypersomnolence and comorbid bipolar disorder or schizophrenia, a clinical dilemma remains: psychostimulants may complicate psychotic symptoms, and antipsychotics can also exert a calming influence on patients. BRX may ameliorate hypersomnolence and psychosis without involving this dilemma. Antidepressants have also been prescribed as an off-label use for cataplexy and sleep paralysis. However, considering that hypersomnolence usually manifests in the second decade of life, there is a non-negligible risk of antidepressant-induced mania. Antidepressant monotherapy has little effect on psychosis. BRX, which affects several receptors as an SDAM, might therefore provide greater advantages for treating individuals with hypersomnolence and psychosis compared to any of the other psychotropic agents.

## Conclusions

In conclusion, we successfully treated IH in a patient with comorbid bipolar disorder by administering BRX. The BRX alleviated not only the patient's manic symptoms but also her hypersomnolence. It is worthwhile to consider the use of BRX for a patient with IH and comorbid bipolar disorder. Further research exploring our present findings is warranted.
